# Health, health-promoting resources, and lifestyle factors among final-semester students in nursing, healthcare, and social work programs, and newly graduated nurses and healthcare and social work professionals - a repeated cross-sectional study

**DOI:** 10.1186/s12912-026-04474-6

**Published:** 2026-03-07

**Authors:** Therese Skogelin, Jenny Hallgren, Inger Ahlstrand, Margaretha Larsson, Ingrid Larsson

**Affiliations:** 1 School of Health and Welfare, Halmstad University, PO Box 823, Halmstad, S-301 18 Sweden; 2 School of Health Sciences, University of Skövde, Skövde, Sweden; 3 School of Health and Welfare, Jönköping University, Jönköping, Sweden

**Keywords:** Early work life, Health-promoting resources, Lifestyle factors, Salutogenic approach, Students

## Abstract

**Background:**

Health promotion in higher education may support sustainable health among future healthcare and social work professionals, who face elevated risk of poor health when entering working life. The aim of this study was to describe associations between general health, health-promoting resources, lifestyle factors, and social factors among final-semester students in nursing, healthcare, and social work programs and newly graduated nurses and healthcare and social work professionals. In addition, the study compared groups reporting excellent/very good general health with those reporting good, fair, or poor health.

**Methods:**

Data were collected through a web-based questionnaire, between 2020 and 2023, which included items on health and lifestyle factors, along with validated instruments measuring health-promoting resources (sense of coherence, salutogenic health, emotional intelligence, occupational balance) and social support. Analyses included chi-square tests, non-parametric tests, and logistic regression.

**Results:**

This study included 343 final-semester students in nursing, healthcare, and social work programs and 115 newly graduated professionals. In the final semester, excellent/very good health was most strongly associated with absence of sleep problems (OR 3.3), no oral tobacco use (OR 2.7), and exercising > 60 min/week (OR 2.8). Higher scores in sense of coherence, emotional intelligence, occupational balance were also linked to better health. Among newly graduated professionals, absence of sleep problems (OR 6.8), daily activity > 150 min/week (OR 3.5), and low alcohol use (OR 2.7) were associated with excellent/very good health. At both time points, groups with excellent or very good general health reported higher scores in sense of coherence, salutogenic health, emotional intelligence, and occupational balance. No significant differences in health or health-promoting resources were found between final-semester students and newly graduated professionals.

**Conclusion:**

Participants reporting excellent or very good health had higher health-promoting resources and better sleep during their final semester in nursing, healthcare, and social work programs and as newly graduated professionals. Neither health nor health-promoting resources changed upon entering work-life, however, this observation should be interpreted cautiously given the study’s limitations and confirmed in future research. Health promotion during higher education and in early work-life may therefore be crucial for sustainable health. As some of the data were collected during the COVID-19 period, results should be interpreted with awareness that pandemic-related circumstances may have influenced lifestyle and perceived health.

## Introduction

The World Health Organization has emphasized a strong need for health promotion among healthcare and social work professionals, e.g., nurses, specialist nurses, radiologists, and social workers, to ensure a healthy and sustainable workforce [1]. The transition from student life to early work-life is a challenging period for newly graduated professionals. Insufficient internal resources and external support during this phase may negatively affect health and influence retention in the profession [2, 3]. Integrating health promotion based on a salutogenic approach into the curriculum has been shown to positively influence students’ health and lifestyle [4]. Health-promoting resources, such as occupational balance, social support, manageability, and meaningfulness, are associated with better general health of students and newly graduated nurses and healthcare and social work professionals. These resources also contribute to favorable lifestyle factors, including sleep quality and exercise [5, 6].

This study is grounded in the salutogenic theory, which focuses on the origin of health rather than disease [7]. Health is viewed as a continuum between health and dis/ease, not a permanent state, and individuals move along this continuum throughout life. Sense of coherence (SOC), comprising comprehensibility, manageability, and meaningfulness, reflects an individual’s capacity to understand, cope with, and find meaning in life stressors [7, 8]. SOC is associated with occupational balance [9] and general health [10] and health-related behaviors [11] among both students [10, 12] and nurses [6], and is considered a reliable predictor of health and all-cause mortality [13]. Strengthening SOC during early adulthood may help mitigate stress-related ill health [8]. In student and early professional populations, these theoretical constructs have practical implications for stress resilience, coping, and health-promoting behaviors, particularly in early stages of professional development [14].

Healthcare and social work professionals face high risks of stress and burnout, which are associated with medical errors at work and an increased risk of a less healthy lifestyle, e.g., high alcohol intake [15]. Healthcare professionals with unhealthy lifestyles are less prone to promote healthy habits to patients and may be perceived as less trustworthy [16]. Entering work-life, nurses and healthcare professionals are at an increased risk of poor health compared to the general population, and many report having a sedentary lifestyle, insufficient sleep, and stress-related symptoms [15–17]. Approximately 25% of healthcare professionals report not engaging in health-promoting behaviors despite awareness of the benefits, with contributing factors including long hours and irregular, inflexible shifts, that are common in these professions [16, 18, 19]. These patterns underscore the importance of understanding which resources support health and lifestyle among students and newly graduated professionals at the start of their careers.

Research has described lifestyle factors, such as alcohol use, diet, tobacco consumption, and physical activity among students across diverse contexts [20–24]. Nursing and social work students often report less healthy lifestyle factors in their final semester, compared to earlier stages of education [25], indicating that the transition to work-life is a vulnerable period. However, limited evidence addresses how health-promoting resources interact with lifestyle factors, during the transition from education to early professional practice. This gap constrains the development of targeted health promotion strategies for this population. The aim of this study was to describe associations between general health, health-promoting resources, lifestyle factors, and social factors among final-semester students in nursing, healthcare, and social work programs and newly graduated nurses and healthcare and social work professionals. In addition, the study compared groups reporting excellent or very good general health with those reporting good, fair, or poor health.

## Methods

### Design

This study used a repeated cross-sectional study, collecting data at two time points during the transition from higher education to early professional life. Most participants were different individuals at each time point. In addition, a small group of participants responded at both time points, which allowed for an exploratory analysis of potential changes over time. The study used data from a project within the Swedish “Health Research in Collaboration” framework, which includes six universities in southern Sweden [26]. The study was reported in accordance with the STROBE (Strengthening the Reporting of Observational Studies in Epidemiology) guidelines for observational research [27].

### Setting and sample

Students enrolled in 2018 in higher education programs for registered nurses, diagnostic radiology nurses, biomedical laboratory scientists, occupational therapists, physiotherapists, registered dental hygienists, and social workers were eligible to participate in the study (*n* = 2283) at baseline. The only exclusion criterion was students who did not speak/read Swedish. No students were excluded [26].

The present study included final-semester students (T6/7), semester 6 or 7 depending on program) in nursing, healthcare and social work programs, and newly graduated nurses and healthcare and social work professionals one year after graduation (Y1). At T6/7, 343 students participated out of 1700 invited, and at Y1, 115 professionals participated out of 1753 invited.

### Data collection

An email with a link to the survey was sent to final-semester students from December 2020 to January 2022, depending on starting time and program, and to the newly graduated nurses, healthcare and social work professionals in spring 2023. The survey was a self-reported web-based questionnaire (esMaker NX3 software) that included questions about their health, sociodemographic characteristics, personal resources, and lifestyle. Three reminders were sent out after the initial email. To ensure that everyone had the opportunity to participate, all eligible individuals were invited at each time point, regardless of whether they had responded to previous surveys [26].

### Instruments

#### General health and well-being

General health and well-being were measured using a single question (“in general, how do you describe your health/well-being?”) rating from excellent (1) to poor (5).

#### Health-promoting resources

The Sense of Coherence 13-item scale (SOC-13) was used to explore the participants’ health-promoting resources. The scale consists of three components: comprehensibility (five items), manageability (four items), and meaningfulness (four items), and answers are provided using a seven-step semantic scale [8]. The scale is validated as a strong indicator of health and psychometric properties, providing a total score that ranges from 13 to 91, with higher scores indicating a higher level of SOC. It can be noted that there is sparse research on what states a good level of SOC for young adults, and Antonovsky described how a person’s SOC is under development until the age of around thirty [8].

The Salutogenic Health Indicator Scale (SHIS) is a 12-item scale that measures the salutogenic aspect of health. It originates from a broad view of health and includes cognitive, physical, and psychosomatic perspectives. The questionnaire stands on the foundation of one question: “How have you been feeling over the past four weeks?” Then continues: “over the past four weeks, I’ve been feeling…”. The answers range from 1 to 6. The instrument has been shown to have high reliability and validity when tested in Sweden [28]. By mistake, only 11 out of 12 items were included in this survey. Unfortunately, the statement about having energy was missing. Therefore, the total score ranges from 11 to 66 points, where higher scores indicate better health.

The Trait Emotional Intelligence Questionnaire Short Form (TEIQue-SF) comprises of 30-items and measures emotional intelligence and the ability to handle stressful situations. The questions are statements, such as “I am able to express my feelings openly”. The response is given on a scale from completely disagree (1) to completely agree (7). The total score is then calculated based on all answers and gives a total score from 1 to 7, where a higher score relates to higher emotional intelligence. The questionnaire has been validated as a reliable psychometric [29].

The Occupational Balance questionnaire (OBQ) was used to capture the experience of occupational balance and focuses on satisfaction with the amount and variation of occupations in life. The OBQ has good content validity, good internal consistency, and sufficient test-retest reliability [30]. The questions are in the style of “When I think of a typical week, I have just enough to do” and “I have a balance between different occupations in my everyday life (employment, home and family chores, leisure occupations, rest and sleep)”. OBQ comprises eleven statements and is measured on a four-step ordinal scale (0–3), with a total score from 0 to 33, where a higher value indicates a higher level of occupational balance [31].

Two questions from the General Nordic Questionnaire for Psychological and Social Factors at Work (QPS Nordic) were used to measure social support: “If needed, can you get support and help with your work from your coworkers?” and “If, needed, are your coworkers willing to listen to problems concerning your work?”. The words coworkers and work were used in the questionnaire for the newly graduated nurses, healthcare and social work professionals, and the questions were modified to adapt for students using the words ‘study’ and ‘study mates’ instead. The response is then provided using a five-step scale rating from never (0) to often (4) [32].

#### Lifestyle factors

Questions about lifestyle factors (diet, exercise, alcohol, tobacco use, sleep) and demographic information were posed according to the Swedish Public Health survey [33].

### Data analysis

The descriptive analysis extracted background statistics. General health was dichotomized to identify those with high general health: responses of excellent or very good were grouped together and compared with responses of good, less good or bad.

Lifestyle factors were dichotomized primarily based on national and international public health recommendations from the National Board of Health and Welfare of Sweden [34] and WHO [35] for physical activity, diet, alcohol, and tobacco use, and on established indicators for sleep problems and sedentary behavior used in public health surveys. The following cut-offs were applied: high-intensity exercise: (> 60–90 min/week = yes; < 30–60 min/week = no), moderate-intensity physical activity, i.e. everyday physical activities: (> 150 min/week = yes; < 150 min/week = no), daily intake of vegetables: yes/no, consumption of alcohol: no or < once per month = no; all other consumption = yes), smoking: yes/no, daily oral tobacco use yes/no, sedentary behavoir (> 10 h/day = yes; < 10 h/day = no), sleep problems (yes/no).

The data were not normally distributed according to the Shapiro-Wilks test, which led to analyses being conducted with non-parametric tests. The comparison between the groups with excellent or very good versus good, fair, or poor general health was analyzed with Mann-Whitney for health and health-promoting resources. For the categorical variables of social support and lifestyle factors, the difference was calculated using Pearson’s chi-square test. Associations to general health were analyzed in a multiple logistic regression where health-promoting resources and lifestyle factors that had significant differences between the two groups with excellent or very good general health and good, fair, or poor general health were simultaneously inserted into the model. Multicollinearity among the independent variables was assessed using variance inflation factors (VIFs), and all VIF values were < 2.8, which is below commonly used thresholds (e.g., 5), indicating no evidence of problematic multicollinearity. A p-value < 0.05 was considered significant.

Furthermore, to capture the change in time, the Wilcoxon signed-rank test was used to compare the health-promoting resources of the final-semester students from nursing, healthcare and social work programs (T6/7) with the newly graduated nurses and healthcare and social work professionals (Y1). The two questions about social support were not included due to the slight difference in the wording and were therefore not considered comparable. Analysis of the subsample comparison over time was based on the participants who responded both as final-semester students and as newly graduated professionals (*n* = 41). All data have been analyzed using IBM SPSS Statistics version 29.0.

## Results

This study included 343 final-semester students in nursing, healthcare, and social work programs, and 115 newly graduated nurses and healthcare and social work professionals. The majority identified as female (Table 1).

**Table 1:** Background characteristics of participants

	T6/7 Total *n* = 343	Y1 Total *n* = 115
Age, years, M(SD)	30.42 (7.67)	34.10 (8.86)
Gender, n		
Female	312	98
Male	30	17
Other	1	0
Rural living area, n	53	20
Living alone, n	72	21
Children at home, n	56	35
Born in Sweden, n	306	98
Program/ profession, n		
Registered nurse	212	70
Diagnostic radiology nurse	3	2
Biomedical laboratory scientist	3	3
Registered dental hygienist	8	2
Occupational therapist	31	9
Physiotherapist	2	5
Social worker	84	24

T6/7 = Final-semester students, Y1 = Newly graduated professionals, M= mean, SD= standard deviation

### Associations with general health

#### Health-promoting resources

Excellent or very good general health was associated with SOC, TEIQue-SF, and OBQ among final-semester students. No health-promoting resources were significantly associated with excellent or very good general health among newly graduated professionals (Table 2).

**Table 2:** Multiple logistic regression of predictive health-promoting resources for excellent and very good general health

Dependent variable excellent/very good GH									
T6/7* Variables	B(SE)	OR	95% CI	p-value	Y1** Variables	B(SE)	OR	95% CI	p-value
Age	-0.056 (0.020)	0.945	0.908–0.984	**0.006**	Age	-0.019 (0.027)	0.981	0.931–1.034	0.471
Sex, *female*	-0.347 (0.488)	0.707	0.272–1.840	0.478	Sex, *female*	0.154 (0.638)	1.166	0.334–4.074	0.810
SOC total	0.045 (0.017)	1.046	1.011–1.082	**0.009**	SOC total	0.047 (0.032)	1.049	0.986–1.116	0.134
SHIS	0.007 (0.019)	1.007	0.969–1.045	0.733	SHIS	-0.046 (0.034)	0.955	0.894–1.020	0.173
TEIQue-SF	0.522 (0.281)	1.737	1.001–3.015	**0.050**	TEIQue-SF	0.483 (0.515)	1.621	0.591–4.451	0.348
OBQ	0.056 (0.023)	1.058	1.012–1.106	**0.013**	OBQ	0.033 (0.046)	1.033	0.943–1.132	0.481

T6/7 = Final-semester students, Y1 = Newly graduated professionals, B= Unstandardized regression coefficient, SE= Standard error, OR= Odds ratio CI= confidence interval, GH= General Health, SOC= Sence Of Coherence, SHIS= Salutogenic Health Indicator Scale, TEIQue-SF= Trait-Emotional Intelligence Questionnaire Short Form, OBQ= Occupational Balance Questionnaire

* R2 = 0,208 (Cox and Snell), 0.282 (Nagelkerke), χ²=68.328, *p* = 0.001

**R2 = 0.221 (Cox and Snell), 0,301 (Nagelkerke), χ²=26,979, *p* = 0.001

#### Lifestyle factors

Not using oral tobacco (snuff), having no sleep problems, and exercising for more than 60 min per week significantly increased the chance of having excellent or very good general health among final-semester students. Having no sleep problems influenced the most with an OR of almost 3.3 times higher chance of excellent or very good general health. Male sex and lower age are also associated with excellent or very good general health among final-semester students. Having no sleep problems, not drinking alcohol more than once a month, and engaging in daily activity for more than 150 min per week were associated with excellent or very good general health among newly graduated professionals. Those with no sleep problems were almost 6.8 times more likely to have excellent or very good general health (Table 3).

### General health

The mean general health for the total sample was 2.70 at T6/7 and Y1. There was a significant difference in general health between the groups with very good or excellent general health versus those with good, poor, or very poor general health, both among final-semester students and among newly graduated professionals (Table 4).

**Table 3:** Multiple regression of predictive lifestyle factors for excellent or very good general health

Dependent variable excellent/very good GH									
T67* Variables	B(SE)	Odds-ratio	95% CI	p-value	Y1** Variables	B(SE)	Odds-ratio	95% CI	p-value
Age	-0.035 (0.17)	0.966	0.934–0.999	**0.042**	Age	0.015 (0.028)	1.015	0.960–1.072	0.608
Sex, *female*	-0.918 (0.453)	0.399	0.164–0.971	**0.043**	Sex, *female*	-0.038 (0.635)	0.936	0.277–3.343	0.953
Sleep problems, *no*	1.194 (0.255)	3.299	2.002–5.436	**< 0.001**	Sleep problems, *no*	1.913 (0.492)	6.772	2.581–17.768	**< 0.001**
Oral tobacco (*snuff)*,* no*	0.979 (0.344)	2.662	1.355–5.229	**0.004**	Daily activity > 150 min/week, *yes*	1.246 (0.470)	3.477	1.384–8.736	**0.008**
Exercises > 60–90 min/week, *yes*	1.028 (0.248)	2.794	1.718–4.545	**< 0.001**	Exercises > 60–90 min/week, *yes*	0.661 (0.481)	1.936	0.754–4.970	0.170
					Alcohol, *no or once a month*	0.981 (0.469)	2.667	1.063–6.690	**0.037**

T6/7 = Final-semester students, Y1 = Newly graduated professionals, B= Unstandardized regression coefficient, SE= Standard error, OR= Odds ratio, CI= Confidence interval, GH= General health

* R2 = 0,156 (Cox and Snell), 0.211 (Nagelkerke), χ²=26.979, p = < 0.001

** R2 = 0.271 (Cox and Snell), 0.368(Nagelkerke), χ²=36.353, p = < 0.001

### Health-promoting resources

There was a significant difference (*p* < 0.001) between those in the group with excellent or very good general health compared to those in the group with good, fair, or poor general health in SOC, SOC components (comprehensibility, manageability, meaningfulness), SHIS, TEIQue- SF, and OBQ, among final-semester students (T6/7) and newly graduated professionals (Y1). There were no significant differences in factors for social support between groups with excellent or very good general health and good, fair, or poor general health, neither in T6/7 nor in Y1(Table 5).

**Table 4:** General health and well-being among final-semester students (T6/7) and newly graduated professionals (Y1)

	T6/7 Total *n =* 343	Excellent/ Very good GH *n =* 133 (39%)	Good/ Fair/ Poor GH *n =* 210	*p*-value*	Y1 Total *n =* 115	Excellent/ Very good GH *n =* 44 (38%)	Good/ Fair/ Poor GH *n =* 71	*p*-value*
GH, M (SD)	2.70 (0.86)	1.81 (0.034)	3.27 (0.037)	< 0.001	2.70 (0.82)	1.82(0.059)	3.24 (0.055)	< 0.001
General well-being M (SD)	2.73 (0.94)	2.16 (0.067)	3.09 (0.059)	< 0.001	2.68 (0.92)	2.02 (0.100)	3.08 (0.098)	< 0.001

T6/7 = Final-semester students, Y1 = Newly graduated professionals, GH= General Health, M= mean, SD= standard deviation

A lower value indicates better health and well-being

*Difference between groups with excellent or very good GH versus good, fair, or poor GH

**Table 5:** Health-promoting resources among final-semester students (T6/7) and newly graduated professionals (Y1)

	T6/7 Total *n =* 343	Excellent/ Very good GH *n =* 133	Good/Fair/ Poor GH *n =* 210	*p*-value*	Y1 Total *n* = 115	Excellent/ Very good GH *n =* 44	Good/Fair/ Poor GH *n =* 71	*p*-value*
SOC Total, M(SD)	61.33(12.02)	66.81 (11.07)	57.93 (11.33)	**< 0.001**	63.77(12.79)	70.02 (9.93)	59.93 (12.90)	**< 0.001**
SOC *comprehensibility*, M(SD)	21.61 (5.54)	23,71 (5.56)	20.28 (5.11)	**< 0.001**	22.69 (5.58)	25.32 (4.96)	21.06 (5.34)	**< 0.001**
SOC *manageability*, M(SD)	18.76 (4.39)	20.70 (3.79)	17.54 (4.31)	**< 0.001**	19.71 (4.52)	21.67 (3.46)	18.50 (4.69)	**< 0.001**
SOC *meaningfulness*, M(SD)	21.01 (3.86)	22.43 (3.49)	20.14 (3.84)	**< 0.001**	21.38 (4.19)	22.98 (3.63)	20.39 (4.22)	**< 0.001**
SHIS total (11 questions), M(SD)	41.90 (10.31)	45,98 (9.32)	39.30 (10.08)	**< 0.001**	43.98 (10.43)	49.09 (6.90)	40.82 (11.02)	**< 0.001**
TEIQue-SF, M(SD)	5.23 (0.69)	5.50 (0.59)	5.06 (0.70)	**< 0.001**	5.23 (0.64)	5.51 (0.52)	5.07 (0.65)	**< 0.001**
OBQ total M(SD)	14.0 (7.44)	17.02 (7.77)	12.04 (6.52)	**< 0.001**	12.82 (6.42)	15.42 (5.62)	11.25 (6.40)	**< 0.001**
Study mates/coworkers support, *yes* % (n)	76.1 (261)	78.9 (105)	74.3 (156)	0.324	96.5 (111)	97.7 (43)	95.8 (68)	0.579
Study mates/coworkers listen, *yes* % (n)	80.5 (276)	82 (109)	79.5 (167)	0.580	94.8 (109)	97.7 (43)	93 (66)	0.264

T6/7 = Final-semester students, Y1 = Newly graduated professionals, GH = General Health, M = Mean, SD = standard deviation, GH = General Health, SOC = Sense of Coherence, SHIS = Salutogenic Health Indicator Scale, TEIQue-SF = Trait-Emotional Intelligence Questionnaire Short- Form, OBQ = Occupational Balance Questionnaire

A higher value indicates better health-promoting resources in all variables

* Difference between groups with excellent or very good GH versus good, fair, or poor GH

### Lifestyle factors

The group with excellent or very good general health was significantly more likely to exercise more than 60–90 min every week, not use snuff, and have no sleep problems among final-semester students (T6/7) compared to those with good, fair, or poor general health. Among newly graduated professionals (Y1), the group with excellent or very good general health was significantly more likely to exercise more than 60–90 min weekly, be moderately active more than 150 min weekly, be non-smokers, and not drink alcohol more than once a month. In addition, the group with excellent or very good general health had a significantly lower incidence of sleep problems compared to those with good, fair, or poor general health after graduation (Table 6).

**Table 6:** Lifestyle factors among final-semester students (T6/7) and newly graduated professionals (Y1)

	T6/7 Total *n* = 343	Excellent/ Very good GH *n =* 133	Good/ Fair/ Poor GH *n =* 210	*p*-value*	Y1 Total *n =* 115	Excellent/ Very good GH *n =* 44	Good/ Fair/ Poor GH *n =* 71	*p*-value*
Smoking, no % (*n*)	92.7(318)	92.5 (123)	92.9 (195)	**0.896**	94,7(108)	100.0 (44)	91.4 (64)	0.046
Oral tobacco (snuff), *no % (n)*	81.0 (278)	87.2 (116)	77.1 (162)	**0.020**	80,0 (92)	77.3 (34)	81.7 (58)	0.565
Exercises > 60–90 min/week, *yes % (n)*	46.6 (149)	60.9 (81)	37.5 (78)	**< 0.001**	58.3 (67)	70.5 (31)	50.7 (36)	**0.037**
Daily activity > 150 min/week, *yes % (n)*	50.6 (173)	55.6 (74)	47.4 (99)	0.136	43.5 (50)	61.4 (27)	32.4 (23)	**0.002**
Sedentary<10 h/day, *yes* % *(n)*	77.8 (267)	79.7 (106)	76.2 (161)	0.510	84.3 (87)	84.1 (37)	84.5(60)	0.952
Sleeping problem, *no % (n)*	56.3 (192)	72.0 (95)	46.4 (97)	**< 0.001**	52.2 (60)	77.3 (34)	36.6 (26)	**< 0.001**
Eating vegetables daily, *yes % (n)*	22.2 (76)	18.0 (24)	24.8 (52)	0.144	31.3 (36)	18.2 (8)	39.4 (28)	**
Alcohol, *no or once a month % (n)*	55.4 (190)	51.1 (68)	58.1 (122)	0.206	50.4 (58)	68.2 (30)	39.4 (28)	**0.003**

T6/7 = Final-semester students, Y1 = Newly graduated professionals, GH = General Health

* Difference between the group with excellent or very good GH versus good, fair, or poor GH

** Not sufficient data for analysis

### Change over time

The mean value of general health did not significantly change between final-semester students (T6/7) and newly graduated professionals (Y1). The mean value for SOC and all three SOC components increased from T6/7 to Y1. SHIS decreased from 35.1 to 33.0. Both TEIQue-SF and OBQ increased slightly. None of the changes were significant (Table 7).

**Table 7:** Changes in general health and health-promoting resources over time for the subsample (participants in both T6/7 and Y1, *n* = 41)

	T6/7	Y1	*p*-value
GH health M (SD)	2.7 (0.086)	2.7 (0.082)	0.492
SOC total, M (SD)	61.3 (12.02)	63.8 (12.79)	0.274
SOC manageability, M (SD)	18.8 (4.39)	19.7 (4.52)	0.193
SOC comprehensibility, M (SD)	21.6 (5.54)	22.7 (5.58)	0.628
SOC meaningfulness M (SD)	21.0 (3.86)	21.4 (4.19)	0.163
SHIS total, M (SD)	35.1 (10.31)	33.0 (10.43)	0.630
TEIQue-SF, total, M (SD)	5.23 (0.69)	5.24 (0.64)	0.259
OBQ total, M (SD)	13.92(6.61)	14.08(7.14)	0.427

T6/7 = Final-semester students, Y1 = Newly graduated professionals, GH= General Health, M= mean, SD= standard deviation, SOC= Sense Of Coherence, SHIS= Salutogenic Health Indicator Scale, TEIQue-SF= Trait Emotional Intelligence Questionnaire Short-Form, OBQ= Occupational Balance Questionnaire

A higher value indicates stronger health-promoting resources in all variables

## Discussion

This study suggests that health-promoting resources may be associated with general health among final-semester students in nursing, healthcare, and social work programs, while no significant associations were observed among newly graduated nurses and healthcare and social work professionals. Furthermore, final-semester students and newly graduated professionals with excellent or very good general health demonstrated better health-promoting resources and were more likely to maintain healthy lifestyle factors such as regular exercise and good sleep, associations that should be interpreted as exploratory rather than causal.

In this study, general health appears stable during the transition from final-semester studies to early work-life. Prior research has shown a positive correlation between education level and reporting excellent or very good health in Western countries [36], whereas another study has suggested that nurses’ general health may decline during the transition and early work-life [3]. In the present study, approximately one-third report excellent or very good health, equivalent to an extensive national population study in Switzerland at roughly the same time [36], suggesting that participants in this study might have neither better nor worse general health than the general public.

The current study shows that excellent or very good general health is associated with SOC, emotional intelligence and occupational balance among final-semester students in nursing, healthcare and social work programs. This aligns with previous research on the health-promoting effect of these health-promoting resources [11, 29]. The mechanisms behind this association between general health, emotional intelligence, and SOC likely include improved capacity to adopt and maintain healthy lifestyle factors and to avoid and manage stressors [8, 37, 38]. SOC has been described as a mediator of health among nursing, healthcare, and social work students and professionals, and it is a known buffer against life stress [13, 39]. The final-semester students in this study also have higher emotional intelligence than the average level reported among higher education students [40], in line with research suggesting that nursing students often have higher emotional intelligence than other students [41]. Interestingly, social support from peers or coworkers and the experience of having peers or coworkers who listen were not significantly higher among final-semester students with excellent or very good general health or newly graduated professionals. Previous research has shown an association between general health and social support in educational settings [42], and coworker support among nurses [17]. The findings from the previous study suggest that, although social support is valuable, individual resources such as SOC, salutogenic health, emotional intelligence, and occupational balance, together with lifestyle factors including sleep, exercise, and daily activity, were associated with excellent or very good general health, whereas social support showed no significant association. The lack of significant associations after graduation may reflect contextual factors in early work-life, such as high job demands and irregular schedules, which can overshadow the influence of individual resources [43]. It is also possible that variability in general health was limited among newly graduated professionals, reducing the likelihood of detecting associations [3]. Other factors not measured in this study, such as personality traits or workplace conditions, may also influence perceived health [13, 17].

In this study, lifestyle factors remained important. The results confirm that sleep, exercise, and daily activity are associated with excellent or very good general health among both final-semester students and newly graduated professionals in nursing, healthcare, and social work. Previous research indicates that these associations are persistent in the general population [36], and sleep regularity has been linked to better general health and higher well-being among healthcare professionals [44]. These findings suggest that adjusting work to allow for regularity and recovery, combined with education on sleep hygiene, may help sustain health and support sustainable work-life despite irregular hours [45].

Health-promoting resources appear relatively stable during the transition into work-life, and no significant differences were observed between final-semester students and newly graduated professionals. Although research in this area is scarce, this could indicate that by the time nursing, healthcare, and social work students reach early work-life, they have developed a foundation of health-promoting resources. This supports the assumption that health promotion in universities is essential for later sustainable work-life [4]. The presence of stable health-promoting resources and unchanged general health during the transition to work-life must be seen as a positive for newly graduated nurses, healthcare and social work professionals, especially given the high demands associated with high levels of stress in early work-life [43]. Preserved health-promoting resources could help manage these demands in a salutogenic manner, as suggested by Antonovsky regarding the pathway between health and SOC [8].

From a practical perspective, universities and training programs could implement structured, salutogenic curricula and skills-based training to strengthen SOC (e.g., stress appraisal and coping training, problem-solving, meaning-making, and coherence-building) [8, 13, 14]. Emotional intelligence may be enhanced through small-group training in self-awareness, emotion regulation, and empathy using simulations and reflective practice [37, 41]. Occupational balance could be supported by workload planning, protected recovery breaks, and coaching in time management and prioritization [9]. Additional measures include peer-support and mentoring schemes [18], education on sleep hygiene, and schedule adjustments to promote regularity and recovery where feasible [44, 45]. These programmatic strategies, combined with supportive policies (e.g., reasonable workload, flexible scheduling, access to counseling and academic advising), may foster sustainable health during late studies and the transition into work.

### Strengths and limitations

This study is a repeated cross-sectional study based on data from the longitudinal “Health care in collaboration” project. To improve research standards and reduce publication bias, a peer-reviewed study protocol was published [26], which is considered a strength. This study is one among several studies within the broader project, some of which have already been conducted [5, 9, 10, 46] while others are still to be carried out. The project provides the opportunity to follow the population over time, offering insights that a single study alone cannot provide.

The repeated cross-sectional design allows for the analysis of potential changes in key variables over time, even though different individuals were included at each time point. Unlike a standard cross-sectional design, which captures a single snapshot and does not allow for temporal comparisons or conclusions about change, this approach provides a broader perspective by enabling the examination of trends or stability across repeated measures. While causal relationships still cannot be established, the repeated design addresses one of the main limitations of traditional cross-sectional studies by incorporating a temporal dimension. Including six universities is a strength, as it provides diversity within the targeted population. The study uses self-report measurements. However, validated instruments with reversed-coded items and variations in response options in the questionnaires reduce the risk of response bias. There could be a risk of self-report bias, given that some people are more likely to fill out questionnaires than others. Missing data were minimal (< 5 cases) and therefore not imputed, but this should be noted as a limitation. Generalizability beyond Sweden may also be limited.

Several methodological limitations should be acknowledged. First, the response rate was low, particularly at Y1 (approximately 6.5%), which introduces a risk of selection bias and limits the representativeness of the sample. Access to data on non-respondents was not available, which prevented a non-response or sensitivity analysis. Therefore, the findings should be interpreted with caution. Low response rates are common in web-based surveys among healthcare professionals, as reported in previous research, but remain an important limitation of this study. Second, the dichotomization of continuous lifestyle variables, although based on established public health recommendations, may have reduced statistical power and led to loss of information, increasing the risk of Type II error. This approach also limits the ability to capture more gradual differences within categories, which should be considered when interpreting the findings. In addition, the number of predictors relative to outcome events in the logistic regression models, particularly for the Y1 sample, may increase the risk of overfitting and affect model stability. The regression findings should be interpreted with caution because some models, especially Y1, had few outcome events per variable. This may reduce precision and model stability. Predictors were selected based on significant bivariate associations to reduce unnecessary variables. Third, the longitudinal subsample was small (*n* = 41), limiting generalizability and reducing power to detect change over time. This may partly reflect methodological limitations, including the small longitudinal subsample, dichotomization of continuous variables, and the omission of one SHIS item, which could have reduced statistical power and influenced non-significant results. Therefore, the conclusion that health-promoting resources do not predict health after graduation may be overstated. Fourth, one item from the SHIS scale was omitted by mistake, compromising construct validity and limiting comparability with previous studies using the full scale. Although Cronbach’s alpha remained acceptable, this limitation should be considered when interpreting SHIS-related findings. Fifth, the study design and measurement tools may not fully capture the theoretical mechanisms proposed by the Salutogenic framework, which should be interpreted with caution. Sixth, data collection overlapped with the COVID-19 pandemic (2020–2023), which may have affected lifestyle behaviors, sleep, mental health, and work conditions. Finally, no adjustments were made for potential confounders such as economic conditions, job stress, work environment, shift schedules, or organizational support, which could have influenced the observed associations.

Despite these limitations, this study explores health-promoting resources and lifestyle factors in final-semester students and newly graduated professionals, an area where previous research is scarce. This study therefore contributes new knowledge and provides an indication of whether these health-promoting resources and lifestyle factors change when students transition into early work-life.

## Conclusion

This study describes general health, health-promoting resources, social factors, and lifestyle factors among final-semester students in nursing, healthcare and social work programs, as well as among newly graduated nurses and healthcare and social work professionals. Participants who reported excellent or very good health had higher health-promoting resources and better sleep, both during their final semester and after graduation. In this sample, no significant changes in health or health-promoting resources were observed during the transition to early work-life. However, this finding should be interpreted cautiously, given the study’s limitations and the exploratory, non‑causal nature of the observed associations, and confirmed in future research. These results may indicate the importance of targeted health promotion during higher education and early work-life to ensure sustainable general health and health-promoting resources throughout a person’s life. These findings highlight the importance of supporting nurses and healthcare and social work professionals in strengthening and maintaining their health-promoting resources during education and early work-life.

## Data Availability

The datasets and materials used and analyzed during the current study are available from the corresponding author upon reasonable request.

## Abbreviations

GH: General health
SOC: Sense of coherence
SHIS: Salutogenic health indicator scale
TEIQue-SF: Trait Emotional Questionnaire Short Form
OBQ: Occupational balance questionnaire
